# The missed chapter on midfoot: Chopart injuries

**DOI:** 10.1007/s11547-024-01905-9

**Published:** 2024-11-04

**Authors:** Bilal Abs, David Ferreira Branco, Axel Gamulin, Paul Botti, Marcello Buzzi, Pierre-Alexandre Poletti, Hicham Bouredoucen, Sana Boudabbous

**Affiliations:** 1 Department of Radiology, Geneva University Hospital, Rue Gabrielle-Perret-Gentil 4, 1205 Geneva, Switzerland; 2 Department of Orthopedic Surgery, Geneva University Hospital, Rue Gabrielle-Perret-Gentil 4, 1205 Geneva, Switzerland

**Keywords:** Chopart, Midfoot, Cone beam computed tomography, Radiography, Epidemiology

## Abstract

**Purpose:**

Midtarsal injuries are often missed at initial presentation which may lead to long-term complications. Nonetheless, radiographs (XR) are used as a primary imaging method. The place of cone beam computer tomography (CBCT) remains unclear in the management of midfoot injuries. The aim of this study was to update imaging findings on traumatic ankle and foot injuries (TAAFI) with CBCT and to assess the sensitivity, specificity and accuracy of XR compared to CBCT for midfoot injuries detections.

**Material and methods:**

All CBCT studies performed due to (TAAFI) that had previous XR were collected for a period of 5 years. They were retrospectively anonymized and analyzed by a radiologist. A second blinded study of XR was made by a second radiologist as a control.

**Results:**

A total of 754 cases were included. Lisfranc and Chopart injuries were detected in 153 (20.2%) and 154 (20.4%) patients, respectively. Lisfranc and Chopart’s lesions combined together were seen in 33 cases (10.7%). A blinded retrospective analysis of XR compared to CBCT shows a sensitivity of 64.9% (56.8–72.4%; 95% CI), a specificity of 95.0% (92.9–96.6%; 95% CI) and an accuracy of 88.9% (86.4–91.0%; 95% CI) for Chopart’s injuries. Regarding Lisfranc, the sensitivity was 62.1% (53.9–69.8%; 95% CI), specificity 99.0% (97.8–99.6%; 95% CI) and accuracy 91.5% (89.3–93.4%; 95% CI).

**Conclusion:**

This cohort study highlights the missed injuries of Chopart on XR and the low association with Lisfranc avulsions. The use of CBCT helps in detecting and assessing midfoot injury.

## Introduction

**Fig. 1:**
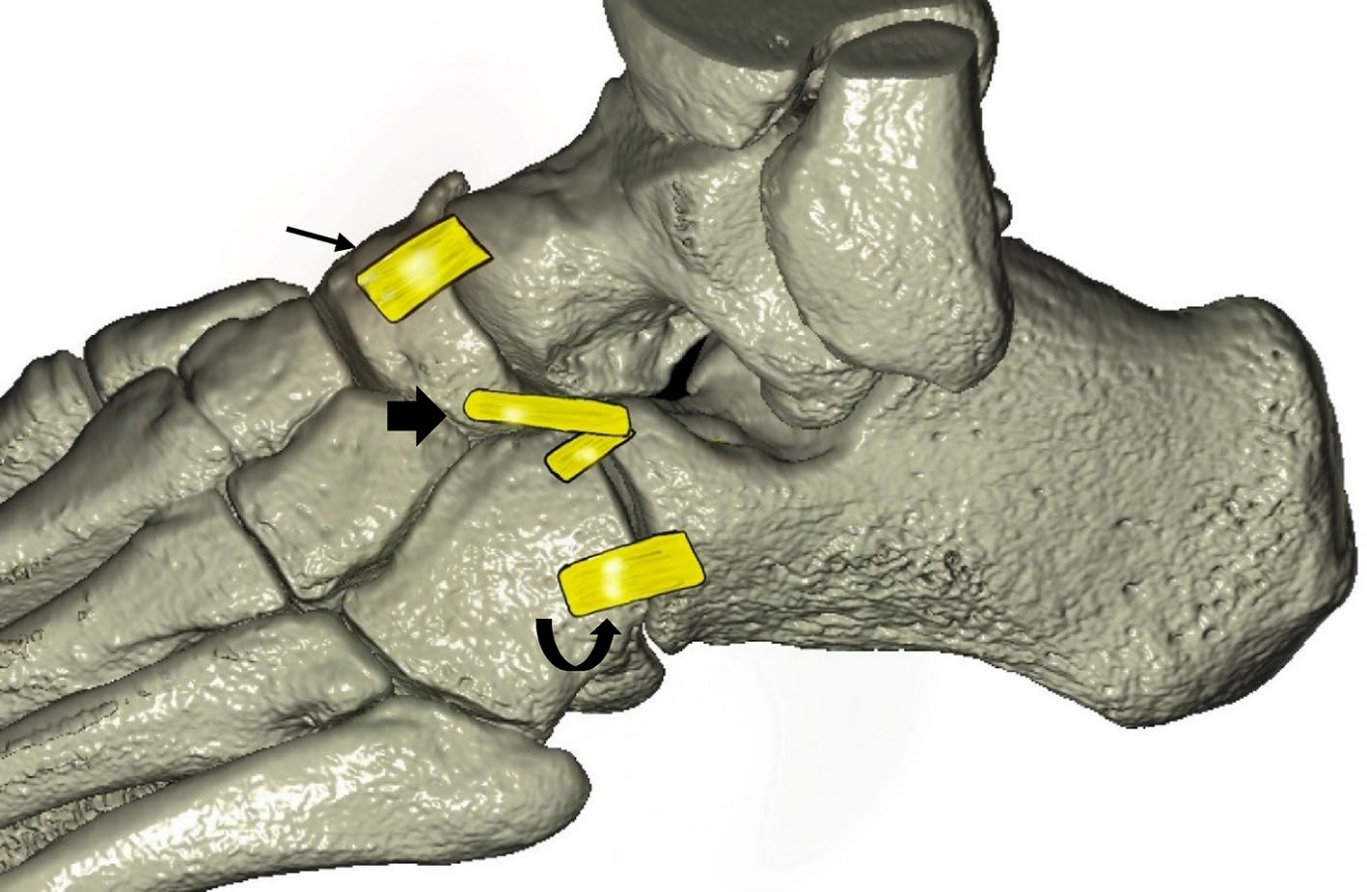
Illustration of the Chopart joint based on VR reconstruction. Thin arrow: dorsal talonavicular ligament. Thick arrow: bifurcate ligament. Curved arrow: dorsal calcaneocuboid ligament

The Chopart joint comprises medially the talocalcaneonavicular joint which is also referred as the talonavicular joint and laterally the calcaneocuboid joint. The talonavicular joint is reinforced by three ligaments: the dorsal talonavicular ligament, the plantar calcaneonavicular ligament and the calcaneonavicular component of the bifurcate ligament [1, 2]. The calcaneocuboid joint contains the calcaneocuboid component of the bifurcate ligament, the dorsal calcaneocuboid ligament, the long and short plantar ligament at the plantar–medial side of the joint (Fig. 1).

According to Court-Brown and al. [3], midfoot fractures are rare with an incidence estimated at 3.6/100,000/year. The most common injury mechanism is midtarsal inversion sprain.

Midfoot injuries are clinically suspected with pain and swelling of the foot with plantar ecchymosis [4] but are often overshadowed or mistaken by lateral collateral ligament injury [5, 6]. As for imaging work-up, the American College of Radiology recommends radiography (XR) with or without weight bearing for initial imaging [7]. Recommendations remain unclear for further assessment after a positive radiography [8]. However, studies show that midtarsal injuries are missed in 22–40% at initial presentation [9, 10] which may lead to long-term complications as acquired posttraumatic flatfoot [10], arthritis, or chronic pain. Regardless, XR are still used as a primary imaging method, most probably due to low radiation, cost and availability [8, 11].

We have witnessed in the past few years the emergence of cone beam computed tomography (CBCT) as a mean to explore extremities. It uses a pyramidal-shaped x-ray bean and flat panel detector that rotates 360° around the patient and offers a high-resolution cross-sectional imaging [12] with or without weight bearing and irradiation doses ranging from 1.3 to 10 folds standard radiography device [13]. The place of CBCT remains unclear in the management of midfoot injuries as, to our knowledge, there is no study in literature evaluating its contribution in the context of acute midfoot injury.

Furthermore, the management of mild Chopart injuries is not consensual in the literature and some reports suggest longer immobilization or surgical treatment for more severe injuries or lesions affecting the medial column, and also for patients needing shorter immobilization time [5, 10, 14, 15].

The objective of this study was to update imaging findings on midfoot injuries and different associations on a 3D imaging technic dedicated to extremities and to assess the current sensitivity, specificity and accuracy of XR for the detection of midfoot injuries compared to CBCT in a large cohort.

### Population

Our study was conducted in a 1900-bed urban academic hospital serving roughly 500′000 inhabitants. According to institutional data, 1% of all emergency admissions are related to acute ankle injuries. Patients admitted to the emergency department with suspicion of midfoot injury after physical examination benefited from XR first. When midfoot injuries were found on XR, the patient would undergo a CBCT for further assessment whenever possible. When the XR was unremarkable and clinical suspicion was moderate or high, CBCT was also performed. If the clinical suspicion was low, no further investigations were carried out. Patients were included prospectively, and imaging (XR and CBCT) was analyzed retrospectively. The protocol for this study was approved by the cantonal ethics committee research (CCER) approval and in accordance with the guidelines of the Helsinki declaration (RCC number 2017-01276).

**Fig. 2:**
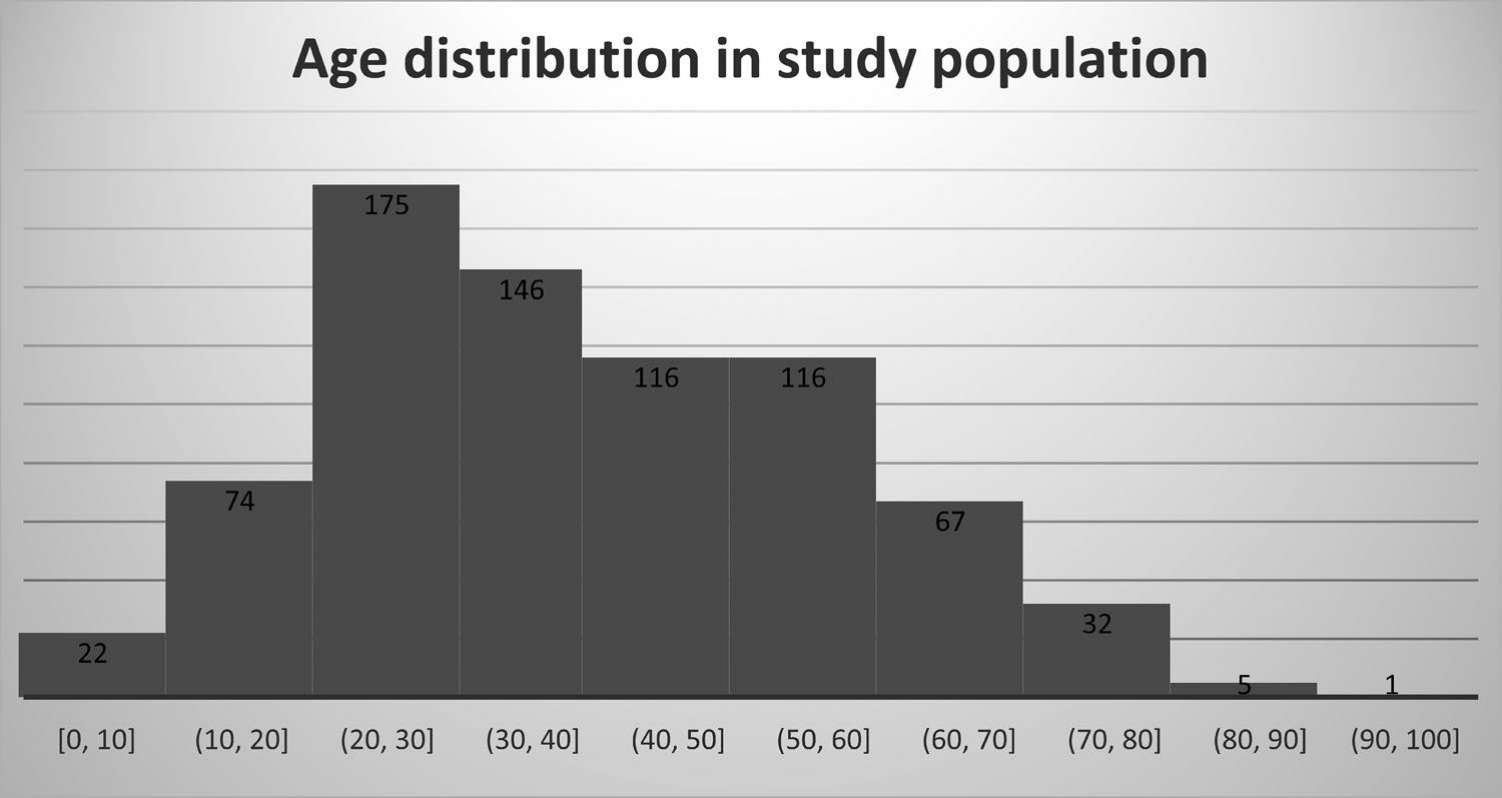
Age distribution in population study

We analyzed all CBCT studies of the foot and ankle performed between March 2018 and December 2022. Of the 1428 patients studied over this period, only patients who underwent CBCT for an acute ankle or foot injury after primary radiography were included in this study; 674 (47%) were excluded for the following reasons: no initial radiography (*n* = 52), chronic trauma follow-up (*n* = 455), osteosynthesis material (*n* = 161), incomplete imaging work-up (*n* = 6). Patients with severe trauma (dislocation, associated vasculonervous lesion, open fractures, bed ridden patients) or polytrauma patients could not benefit from CBCT and therefore did not meet the inclusion criteria. A total of 754 patients met the inclusion criteria, of which 429 were men and 325 were women with a mean age of 40.8 years and an age distribution as visualized in Fig. 2.

### Imaging analysis

**Fig. 3:**
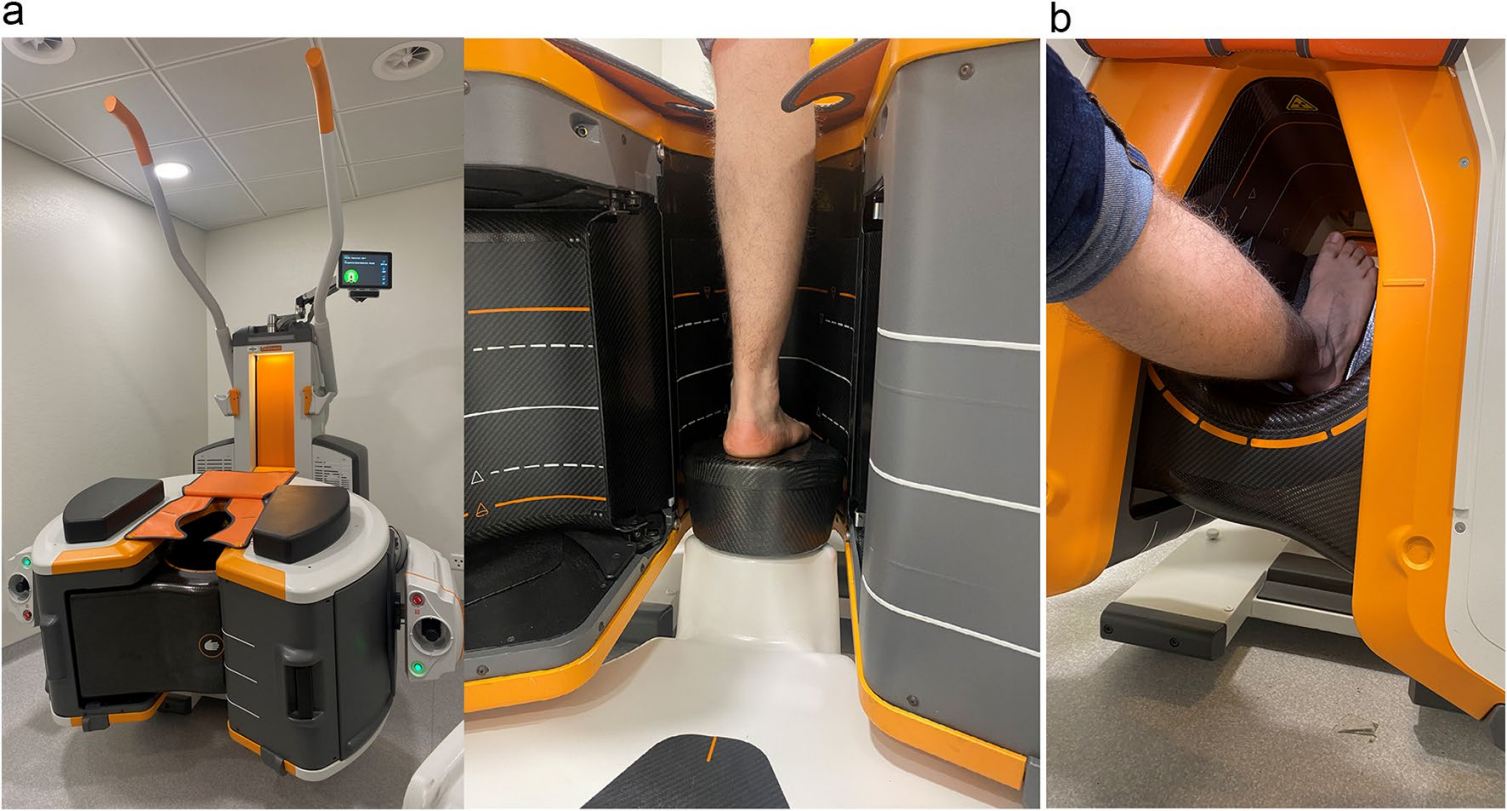
**a** CBCT in position for acquisition in standing position **b** or acquisition in a seated position if standing is not possible

The CBCT (OnSight, Carestream Health, Rochester, New York, US) displays a gantry featuring a 58 cm aperture and movable table (Fig. 3). Weight bearing images were acquired whenever possible. The acquisition parameters for the ankle are summarized in Table 1. All images were reconstructed in coronal and sagittal plane, with bone Kernel with a slice thickness of 0.26 mm (window width: 1500; window level: 3000) using the first-generation model-based iterative reconstruction. Prior to acquisition, two scouts were performed (antero-posterior and antero-lateral view). XR included, depending on the initial clinical suspicion of midfoot views (postero-anterior, oblique, lateral views) and/or ankle view (postero-anterior, lateral). Whenever a Lisfranc injury was suspected, a weight bearing view was added. Images were anonymized and randomized for each modality. They were stored in PACS (Osirix®, Pixmeo SARL, Bernex, Switzerland). A radiologist with 7 years experience reads first the XR and then filled a form retrieving avulsion of the Chopart joint (separated in dorsal talonavicular ligament, bifurcate or dorsal calcaneocuboid ligament avulsion), a Lisfranc injury or ankle avulsion fracture (separated in medial, lateral, or posterior malleolus). All other fractures were noted separately. The same radiologist read after a month time laps all CBCT filling the same form randomly. A second blinded lecture of XR was made by an independent radiologist with 6 years of experience.

**Table 1:** Scanning parameters of the CBCT (OnSight, Carestream Health, Rochester, New York) and digital radiography (DR) (Siemens, ISIO and Philips, DigitalDiagnost)

Parameters	CBCT	DR
Energy	80kVp	50kVp
Current	5 mA	3.2mAs
FOV	216 × 216 mm	
Matrix	884 × 884	
Isotropic voxel size	0.26 mm	
Rotation time	25.18 s	
Exposure time	21 s approximately	
Scan rotation angle	216.5°	
CTDI (indicated)	3.14 mGy (16 cm phantom)	
Focus detector distance		120 cm

### Statistical analyses

MedCalc (MedCalc Software Ltd, Ostend, Belgium) was used for statistical analyses. Descriptive analyses for incidence of midfoot injuries were reported for XR and CBCT. To estimate inter- and intra-observer reliability values, weighted Cohen's Kappa for ordinal data was used for normally distributed data while 95% confidence intervals were calculated for each value. Values were considered ≤ 0 as indicating no agreement, 0.01–0.20 as none to slight, 0.21–0.40 as fair, 0.41–0.60 as moderate, 0.61–0.80 as substantial and 0.81–1.00 as almost perfect agreement. Contingency tables were used to assess diagnostic performance of XR in detecting midtarsal injuries. Then, accuracy for XR was assessed taking subsequent CBCT as imaging reference standard for injuries findings. Sensitivity, specificity as well as positive predictive value (PPV) and negative predictive value (NPV) were calculated.

## Results

Our study showed high inter-observer agreement (Table 2) for the XR detection of Lisfranc and Chopart fractures. In view of the high inter-observer reliability, intra-observer reliability between modalities was calculated according to the measurements of the most experienced observer with substantial agreement (0.60–0.80) between modalities. The contingency tables were calculated based on the results of the same most experienced observer.

**Table 2:** Inter-rater agreement between observers on XR and intra-rater agreement between XR and CBCT based on the most experienced observer

	Lisfranc		Chopart	
Inter-rater agreement on XR between observers 1 and 2	Weighted Kappa	0.89180	Weighted Kappa	0.78,622
	Standard error	0.02443	Standard error	0.03022
	95% CI	0.84,392–0.93968	95% CI	0.72,698–0.84,546
Intra-rater agreement between XR and CBCT for observer 1	Weighted Kappa	0.69,955	Weighted Kappa	0.63,620
	Standard error	0.03457	Standard error	0.03620
	95% CI	0.63,179–0.76,730	95% CI	0.56,524–0.70,716

### Descriptive analysis

Of the initial 754 patients, 547 had at least one fracture. The number of fractures by anatomical region is shown in Table 3. Lisfranc lesions were detected in 153 patients (20.2%), and 154 patients had involvement of at least one injury site on the Chopart (20.4%). Overall, the mean age of patients with a Chopart lesion was 39.9 years old, 49.7% of whom were women. Among the patients with a Chopart lesions, 92 of them (59.7%) had talonavicular avulsion, 117 (75.9%) had calcaneonaviculocuboid avulsion, and 71 (46.1%) had dorsal calcaneocuboid lesions.

**Table 3:** Listing of the fractures found on CBCT

Localization	Lisfranc	Dorsal talonavicular	Bifurcate	Dorsal calcaneocuboid
Total number	153	92	117	71

### XR performance

Blinded retrospective analysis of prior XR compared with CBCT shows 54 patients had a Chopart injury on CBCT that was not diagnosed on XR, for a sensitivity of 64.935% (56.842–72.441%; CI 95%), a specificity of 95.000% (92.939–96.601%; CI 95%) and an accuracy of 88.859% (86.394–91.016%; CI 95%); 58 patients had an undiagnosed Lisfranc injury on XR with a sensitivity of 62.092% (53.903–69.802%; CI 95%), a specificity of 99.002 (97.840–99.633%; CI 95%) and an accuracy of 91.512% (89.290–93.402%; CI 95%). Overall, for detection of Lisfranc and Chopart avulsions with XR, we obtained a sensitivity of 63.52% (57.86–68.91%; CI 95%), a specificity of 97.09% (95.99–97.95% CI 95%) and an accuracy of 90.41% (88.84–91.84%; CI 95%).

**Fig. 4:**
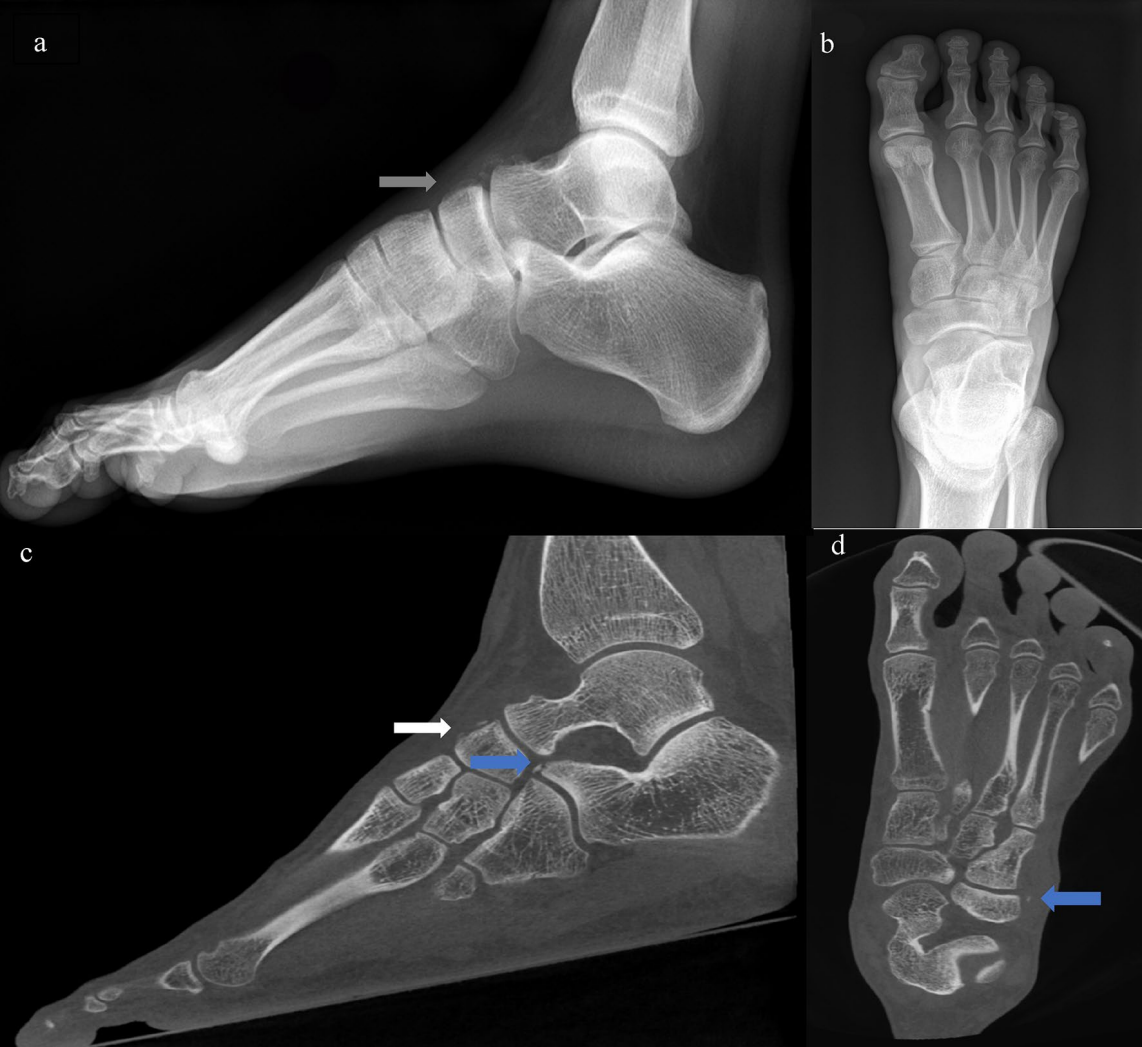
Standard XR **a** sagittal and **b** axial in a patient with multiple Chopart involvement not visible on the XR and found on CBCT’s multiplanar reformation (MPR) **c** sagittal and **d** axial. The gray arrow shows a talonavicular fracture/avulsion visible on XR. The white arrow points to a talonavicular fracture/avulsion visible on XR and CBCT. The blue arrow shows a fracture/avulsion of the anterior calcaneal process and calcaneocuboid ligament avulsion not visible on XR

**Fig. 5:**
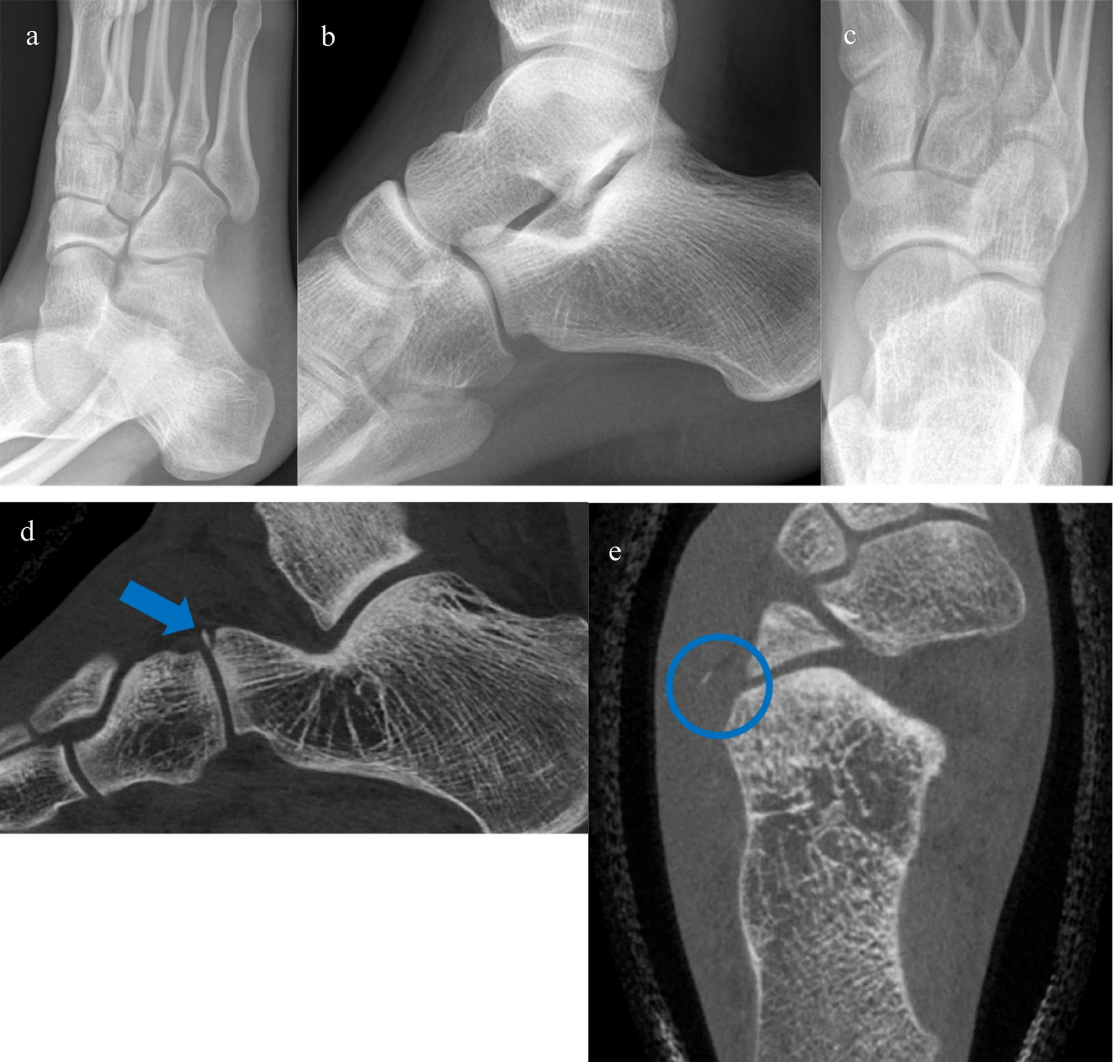
A 17-year-old patient with persistent pain after ankle traumatism with no visible fracture on XR **a** oblique, **b** lateral and **c** antero-posterior views. CBCT of the same patient **d** lateral and **e** axial views. The arrow points to an anterior calcaneal process fracture. The circle shows a dorsal calcaneocuboid fracture

**Fig. 6:**
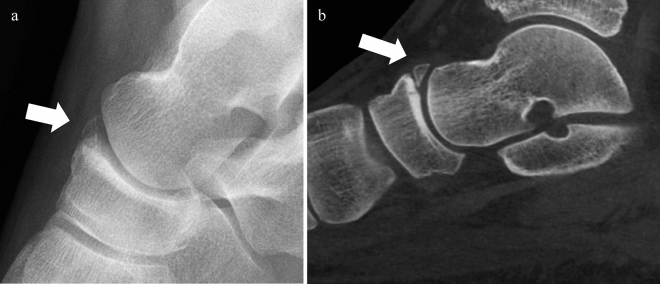
Example of false positive: a 44-year-old patient with an acute trauma of the ankle. The XR on **a** lateral view concluded to an acute talonavicular avulsion as no clear corticalization of the bone fragment was seen. CBCT on **b** lateral plane of the same patient: fragment related to a chronic avulsion of talonavicular ligament without acute pattern (white arrow)

A total of 118 patients with a positive XR for a Chopart lesion had additional lesions found on CBCT (76.6%) (Fig. 4). The most common occult Chopart fracture on XR was the calcaneonaviculocuboid ligament one (Figs. 4 and 5) with a sensitivity of 22.222% (15.059–30.841%; CI 95%). Other common occult fractures involved the dorsal calcaneocuboid ligament, with a sensitivity of 33.803% (11.997–46.007%; CI 95%). The most frequent causes of false-positive Chopart's lesions were fracture sequelae or accessory bones (Fig. 6). The combination of both Lisfranc and Chopart lesions was seen in only 33 cases (10.7%) when only one fracture/avulsion site on the Chopart joint was considered, and in only 2.25% when all three Chopart sites were involved. Finally, malleolar lesions were detected in 26 and 42 cases, respectively, for the medial and lateral sides, with no significant association with Lisfranc or Chopart lesions (0.66% and 1.32%, respectively). Severe ankle and foot injuries such as dislocation were excluded from this study, as were obvious malleolar fractures.

## Discussion

This study highlights that 3D extremity imaging use for mild ankle traumatism provides better detection of Chopart injuries than XR. Our results show that approximatively one third of midfoot fractures were not detected in initial XR. In addition, cross-sectional imaging was useful for the detection of additional fracture site for Chopart injury. Indeed, 76.6% of patients with positive X-ray had additional fractures.

According to Court-Brown [3], the incidence of midfoot fractures was estimated at 3.6/100′000 inhabitant per year, of which 62.9% were avulsion fractures. Our study found an incidence of 6.5/100′000/year of mild Chopart’s lesions and an incidence of 12.9/100′000/ year of Lisfranc and/or Chopart lesions, confirming an under-diagnosis of midfoot fractures as previously suggested by Almeida et al. [8] and Haapamaki et al. [11]. This difference is observed even though our hospital is not the only trauma center in the region and, as mentioned previously, there is an under-representation of more severe injuries in our study directly managed surgically with no CBCT beforehand. This difference may be explained by greater detection of these injuries in our center due to the accessibility to CBCT and due to a higher resolution resulting in a better detection of smaller avulsions than multidetector computed tomography (MCDT) [12, 13].

XR were also less specific for Chopart's lesions. False positives were mainly due to the presence of fracture sequelae or accessory bone (e.g., os supranaviculare, os subtalare, os calcanei secundarium) [16]. High-resolution cross-sectional imaging has made it easy to correct this error by showing well corticalized edges for non-acute lesions, thus avoiding unnecessary prolonged immobilization for patients.

Almeida et al. and Hirschmann et al. [8, 17] found that calcaneocuboid injuries (including bifurcate and dorsal calcaneocuboid ligaments) were the most frequently affected column in Chopart's injury. Their result can be explained from a biomechanical point of view as inversion trauma is the most frequent mechanism; distraction injury to the calcaneocuboid should occur first. In our study, bifurcate avulsion was the most common, followed by talonavicular avulsion and then dorsal calcaneocuboid avulsion. Differentiation between a lesion of the calcaneonavicular component (part of the talonavicular joint [1]) and the calcaneocuboid component (part of the calcaneocuboid joint [1]) of the bifurcate was not always possible on CBCT (such as avulsion of the anterior process of the calcaneus), and we did not separate lesions of these two components. Despite differences in classification, our results are consistent with previous studies.

In our practice, for mild ankle and foot strains, we frequently encounter Chopart injuries. The routine use of CBCT changed our daily practice because it has the best results for the analysis of bone structures of the ankle in comparison with MDCT and MRI [18], has a relatively low irradiation doses of only 1.3 fold compared to XR in best cases [13], and allows weight bearing imaging for foot and ankle (Fig. 3b). CBCT also holds the advantage over XR of not being dependent on patient good positioning, which may be difficult to obtain in the context of emergency. Therefore, CBCT can be considered as an alternative to radiography and as a primary imaging technique due to its relatively low cost and radiation exposure.

However, the main constraint remains the limited availability of CBCT. It is a very specific device that can only be used for imaging extremities. As a result, only large medical centers with a sufficient number of orthopedic patients are equipped with CBCT, and even fewer have it permanently available. XR are much more widely available, allowing rapid access to imaging, which can be interpreted by qualified clinicians, when no radiologist is available on site, speeding up patient flow.

Although our study focused on CBCT, our results are close to those obtained in the literature for CT as demonstrated by Almeida et al. [8]. Initial assessment by CT could be considered as a suitable alternative to CBCT in non-equipped institutions despite a higher effective radiation dose [13] because of the significant improvement in detection and assessment of midfoot lesions [8, 11], leading to more appropriate treatment. MRI could also be considered as an alternative due to its better representation of soft tissue lesions [5, 19] and the absence of ionizing radiation. However, its limited availability, high cost and poorer representation of bone lesions may be a barrier to its widespread use. In addition, the statistically significant advantage of MRI over cross-sectional imaging and radiography remains to be demonstrated [8]. Ultrasound is also an interesting tool for diagnosing Chopart's sprain [6], but its use is limited in the absence of a qualified ultrasonographer.

The strength of our study is that it is, to our knowledge, one of the largest recent studies conducted with confirmed cases of mild Chopart's injuries and the only study comparing XR to CBCT. Our study misrepresented high velocity trauma, whereas Haapamaki et al. [11] clearly stated that their study overestimated high energy trauma because their study population consisted of patients treated at a level 1 trauma center and patient with more simple injuries was excluded. This main difference explains why our sensitivity for the detection of Lisfranc and Chopart injuries was in the lower limit of results found in the literature [20, 21], why our study did not find any dislocation of the foot and low associated injury between midfoot and ankle injuries. However, this study did find mild trauma to the ankle associated with approximately 1.32% of Chopart lesions and 0.66% of Lisfranc lesions and suggests that imaging should be completed with MDCT or CBCT.

In our institution, the additional findings of CBCT in case of foot and ankle strains lead us to differentially treat these lesions according to the detailed knowledge of the anatomy of Chopart involvement. The absence of bony lesions on CBCT indicates a functional treatment with eventual short-term immobilization and weight bearing restriction (7–10 days) depending on the related pain and ability to bear weight. Extra-articular bony avulsions involving only one column are treated within 4 (lateral column) to 6 (medial column) weeks by immobilization in a short-leg cast without weight bearing followed by progressive weight bearing without immobilization over the next 4 weeks. The duration of immobilization and weight bearing restriction is prolonged to 8 weeks if both columns (lateral and medial) are involved. The same protocol is used for non-displaced intra-articular fractures. When a displaced intra-articular fracture or impaction is highlighted by CBCT, internal fixation might be indicated, depending on the size and localization of the bony lesion. Lateral column fractures might still be treated conservatively as related malunions or non-unions are generally functionally more forgiving than medial column fracture malunions or non-union, whereas medial column fractures usually require anatomical reduction and rigid fixation. Additionally, mainly plantar intra-articular fractures or impactions are generally not accessible to fixation. Finally, articular dislocations are surgically treated using anatomical reduction and rigid fixation techniques as well as arthrorysis constructs in some cases. This institutional protocol was developed over the years and updated following the findings of the review published in 2017 by Kutaish et al. [10]. The authors feel that a detailed anatomical knowledge of traumatic lesions of the Chopart brought by a CBCT examination is a necessary and low radiating step to choose the right treatment for each patient with such a condition.

This study was not designed to assess the prognosis of missed midfoot avulsion on radiography. Studies show that early recognition and restoration of normal alignment of both medial and lateral columns are important for a better outcome [9, 22]. The latest larger study assessing the prognosis of ankle sprain injury [23] highlighted that there is insufficient evidence that imaging findings may be used as prognostic factor. A similar systematic review for the midfoot would be interesting for the evaluation of the importance of missed midfoot injuries on XR.

## Conclusion

In conclusion, midfoot Chopart’s injuries are underdiagnosed and their incidence is underestimated in mild midfoot and ankle trauma due to the low sensibility and specificity of XR. Therefore, a low-radiation cross-sectional imaging technique such as CBCT should be considered as primary imaging for detection and characterization of a midfoot lesion leading to a better guidance for orthopedic treatment and prevention of arthritis and long-term instability [9, 24, 25].
